# Recurrence after low-dose radioiodine ablation and recombinant human thyroid-stimulating hormone for differentiated thyroid cancer (HiLo): long-term results of an open-label, non-inferiority randomised controlled trial

**DOI:** 10.1016/S2213-8587(18)30306-1

**Published:** 2019-01

**Authors:** Hakim-Moulay Dehbi, Ujjal Mallick, Jonathan Wadsley, Kate Newbold, Clive Harmer, Allan Hackshaw

**Affiliations:** a Cancer Research UK & UCL Cancer Trials Centre, UCL Cancer Institute, University College London, London, UK; b Freeman Hospital, Newcastle upon Tyne, UK; c Weston Park Hospital, Sheffield, UK; d Royal Marsden Hospital, Sutton, UK; e Independent Doctors Federation, London, UK

## Abstract

**Background:**

Two large randomised trials of patients with well-differentiated thyroid cancer reported in 2012 (HiLo and ESTIMABL1) found similar post-ablation success rates at 6–9 months between a low administered radioactive iodine (^131^I) dose (1·1 GBq) and the standard high dose (3·7 GBq). However, recurrence rates following radioactive iodine ablation have previously only been reported in observational studies, and recently in ESTIMABL1. We aimed to compare recurrence rates between radioactive iodine doses in HiLo.

**Methods:**

HiLo was a non-inferiority, parallel, open-label, randomised controlled factorial trial done at 29 centres in the UK. Eligible patients were aged 16–80 years with histological confirmation of differentiated thyroid cancer requiring radioactive iodine ablation (performance status 0–2, tumour stage T1–T3 with the possibility of lymph-node involvement but no distant metastasis and no microscopic residual disease, and one-stage or two-stage total thyroidectomy). Patients were randomly assigned (1:1:1:1) to 1·1 GBq or 3·7 GBq ablation, each prepared with either recombinant human thyroid-stimulating hormone (rhTSH) or thyroid hormone withdrawal. Patients were followed up at annual clinic visits. Recurrences were diagnosed at each hospital with a combination of established methods according to national standards. We used Kaplan-Meier curves and hazard ratios (HRs) for time to first recurrence, which was a pre-planned secondary outcome. This trial is registered with ClinicalTrials.gov, number NCT00415233.

**Results:**

Between Jan 16, 2007, and July 1, 2010, 438 patients were randomly assigned. At the end of the follow-up period in Dec 31, 2017, median follow-up was 6·5 years (IQR 4·5–7·6) in 434 patients (217 in the low-dose group and 217 in the high-dose group). Confirmed recurrences were seen in 21 patients: 11 who had 1·1 GBq ablation and ten who had 3·7 GBq ablation. Four of these (two in each group) were considered to be persistent disease. Cumulative recurrence rates were similar between low-dose and high-dose radioactive iodine groups (3 years, 1·5% *vs* 2·1%; 5 years, 2·1% *vs* 2·7%; and 7 years, 5·9% *vs* 7·3%; HR 1·10 [95% CI 0·47–2·59]; p=0·83). No material difference in risk was seen for T3 or N1 disease. Recurrence rates were also similar among patients who were prepared for ablation with rhTSH and those prepared with thyroid hormone withdrawal (3 years, 1·5% *vs* 2·1%; 5 years, 2·1% *vs* 2·7%; and 7 years, 8·3% *vs* 5·0%; HR 1·62 [95% CI 0·67–3·91]; p=0·28). Data on adverse events were not collected during follow-up.

**Interpretation:**

The recurrence rate among patients who had 1·1 GBq radioactive iodine ablation was not higher than that for 3·7 GBq, consistent with data from large, recent observational studies. These findings provide further evidence in favour of using low-dose radioactive iodine for treatment of patients with low-risk differentiated thyroid cancer. Our data also indicate that recurrence risk was not affected by use of rhTSH.

**Funding:**

Cancer Research UK.

## Introduction

Thyroid cancer incidence has almost tripled in the USA in the past few decades, from 4·9 cases per 100 000 individuals in 1975 to 14·3 per 100 000 in 2009.^1^ Similar trends are observed in the UK,^2^ where incidence has more than doubled since the 1970s and is projected to rise by 74% between 2014 and 2035. Most cases are differentiated thyroid cancer,^3^ with high 5-year survival rates of 90–95%.^2^, ^4^

Most patients with differentiated thyroid cancer have total or near-total thyroidectomy followed by radioactive iodine (^131^I) ablation and thyroid-stimulating hormone suppression therapy. Results of two large non-inferiority trials (HiLo^5^ and ESTIMABL1^6^) published in 2012 have shown that a low administered radioactive iodine dose (1·1 GBq) for remnant ablation is as effective as the high dose (3·7 GBq), which was the standard for many years; the primary outcome was ablation success at 6–9 months after surgery. One of these trials, the HiLo trial, enrolled low-risk and intermediate-risk patients with differentiated thyroid cancer. The low dose was associated with a lower rate of adverse events than the high dose, a shorter stay in hospital isolation, and lower health-care costs.^5^, ^7^ Although both trials provided definitive evidence that the low dose had a similar ablation success rate as the high dose, whether this short-term outcome translates into longer-term effects on recurrence risk remained an open question.

**Research in context**

**Evidence before this study**

For patients with low-risk and intermediate-risk differentiated thyroid carcinoma, two large non-inferiority randomised trials (HiLo and ESTIMABL1) published in 2012 showed that a low administered radioactive iodine (^131^I) dose (1·1 GBq) had a similar ablation success rate after 6–9 months as the standard high dose (3·7 GBq) for thyroid remnant ablation. The effect of radioactive iodine dose on the risk of recurrence was only reported in observational studies. Most studies comprised low-risk patients, comparing those treated with or without radioactive iodine ablation. Three literature reviews, covering studies published until 2014, concluded that there is little or no difference between radioactive iodine and no radioactive iodine in thyroid cancer recurrence or mortality risk in low-risk patients. We searched PubMed for studies published in English between January, 2008, and December, 2017, comparing low-dose and high-dose radioactive iodine in low-risk and intermediate-risk patients. We used the keywords “differential thyroid cancer/carcinoma”, “radioiodine ablation”, and “low/high dose”. We identified five studies, with sample sizes ranging between 176 and 970 and median follow-up of at least 5 years, suggesting that there is no increased recurrence risk in patients treated with low-dose radioactive iodine compared with high-dose radioactive iodine. There had been no data on recurrences from randomised trials until the follow-up study from ESTIMABL1, which indicated that recurrence was not related to the radioactive iodine dose.

**Added value of this study**

In the HiLo trial, which comprised 434 patients, 21 clinically confirmed recurrences were seen during a median follow-up of 6·5 years. The risk of thyroid cancer recurrence was not higher among patients who had 1·1 GBq than among those who had 3·7 GBq ablation. Similarly, the recurrence risk was not materially higher among patients prepared for ablation with recombinant human thyroid-stimulating hormone than among those prepared with thyroid hormone withdrawal. We noted that several recurrences were seen after 5 years, which is worth consideration by clinicians treating patients for thyroid cancer, given that these patients are typically discharged from the clinic at 5 years in some centres. The few secondary primary cancers seen were balanced between the low-dose group and the high-dose group.

**Implications of all the available evidence**

Data from two randomised trials (HiLo and ESTIMABL1) and recent observational studies provide reliable evidence to further assure patients and clinicians of the benefits of using the low administered dose of radioactive iodine in selected patients. Guidelines can also now be strengthened on the basis of this evidence.

US and UK guidelines^8^, ^9^ now recommend 1·1  GBq in selected low-risk patients, but these guidelines and others have commented on the lack of long-term follow-up data from randomised trials in relation to recurrence rates.^10^ Available evidence for the effect of radioactive iodine dose on long-term recurrence rates comes from observational studies and only one randomised clinical trial, ESTIMABL1.^11^ Observational evidence comes primarily from studies in low-risk patients, comparing those treated with or without radioactive iodine ablation. Two systematic reviews of studies published up to 2008^12^, ^13^ concluded that there was little or no difference between the low dose and high dose in either thyroid cancer recurrence or mortality risk, which was subsequently confirmed by a systematic review^14^ of studies published between 2008 and 2014. Five recent studies have compared low-dose and high-dose radioactive iodine in patients at low and intermediate risk.^15^, ^16^, ^17^, ^18^, ^19^ They also provide no evidence of an increase in recurrence risk among patients treated with low-dose radioactive iodine. In the largest of these studies (comprising 970 patients with a median follow-up of 5 years), the crude proportion of patients with a recurrence was lower in those who had a dose of 3·0 GBq or lower (four [2·6%] of 153 patients) than in those who had a dose higher than 3·0 GBq (61 [7·5%] of 817), even though the low-dose group had more patients with positive nodes (p=0·34 from the multivariable analysis). The investigators of the ESTIMABL1^11^ randomised study recently reported their findings, based on 726 patients after a median follow-up of 5·4 years, which showed that recurrence risk was not influenced by the ablation dose.

The HiLo study aimed to investigate whether low-dose radioactive iodine could be used instead of the high dose in low-risk or intermediate-risk patients, and whether patients could receive recombinant human thyroid-stimulating hormone (rhTSH) before ablation instead of thyroid hormone withdrawal. Here, we report long-term follow-up data of patients in the HiLo trial to examine recurrence rates.

## Methods

### Study design and patients

HiLo was an open-label, non-inferiority, randomised controlled factorial trial done at 29 centres in the UK. The methods, study protocol, and results for the primary outcome^5^ have been published. Approval for the study was obtained from the national research ethics panel of the UK. All patients provided written informed consent to participate in the study.

Eligible patients were aged 16–80 years, with performance status of 0 to 2, histological confirmation of differentiated thyroid cancer requiring radioactive iodine ablation, tumour stage T1 to T3 with the possibility of lymph-node involvement but no distant metastasis and no microscopic residual disease (ie, N0, NX, N1, and M0 in the tumour-node-metastasis [TNM, 6th edition] staging system), and one-stage or two-stage total thyroidectomy with or without prophylactic central lymph-node dissection.

Exclusion criteria were the presence of aggressive malignant variants, anaplastic or medullary carcinoma, pregnancy, severe coexisting conditions, previous cancer with limited life expectancy, previous ^131^I or ^123^I pre-ablation scanning, and previous treatment for thyroid cancer except for surgery.

The full list of inclusion and exclusion criteria is available in the appendix.

### Randomisation and masking

Patients were randomly assigned to low-dose (1·1 GBq) or high-dose (3·7 GBq) radioactive iodine remnant ablation, each with either rhTSH (thyrotropin alfa [Thyrogen], provided by Genzyme) or thyroid hormone withdrawal, in a 1:1:1:1 randomisation ratio. Randomisation was stratified for centre, tumour stage, and nodal stage, and was done centrally (thus avoiding allocation concealment bias). This was an open-label trial, so it was not possible to mask the clinical staff or assessors.

### Procedures

Patients were given an intramuscular injection of 0·9 mg rhTSH on each of the 2 days before ablation. In the thyroid hormone withdrawal group, thyroxine (200 μg per day as the average dose) was discontinued 4 weeks before ablation in 11 patients, and triiodothyronine (60 μg per day as the average dose) was discontinued for 2 weeks in 204 patients (the drug used was missing for four patients). Radioactive iodine ablation was administered 1 month to 6 months after surgery, at a dose of 1·1 GBq or 3·7 GBq depending on the study group allocation. Discharge from hospital isolation took place after satisfactory assessment of radiation risk and clinical conditions.

Following the assessment scan at 6–9 months after radioactive iodine ablation, all patients were meant to be seen in the clinic annually, according to the National Institute for Health and Care Excellence (NICE) guidance on head and neck cancers^20^ and British Thyroid Association (BTA) guidelines.^9^ Participating hospitals were actively contacted at least annually for patient outcomes. Data on adverse events were not collected during follow-up.

### Outcomes

This report focuses on recurrence, which was a pre-specified secondary outcome of the HiLo trial, and was defined as any evidence of (usually structural) disease identified during follow-up. Patients were diagnosed according to national standards using serum thyroglobulin and thyroglobulin antibody, ultrasound, fine needle aspiration cytology, radioactive iodine scans, PET-CT scans, and MRI, as applicable. These tests were used either together or one method (eg, serum thyroglobulin) could lead to further investigations (eg, fine needle aspiration). All patients had either fine needle aspiration or sensitive imaging (PET-CT or radioactive iodine scans). Four patients were diagnosed by both serum thyroglobulin and radioactive iodine, which might indicate some biochemical recurrences. Recurrence could also include persistent disease, reflecting incomplete surgery or ablation failure. However, confirmation of persistent disease was difficult because ultrasound was not routinely used in the UK at the time of the trial as part of the 6–9-month assessment after ablation, so it was not possible to reliably ascertain whether a patient had a structural complete response (and was therefore disease-free) in order to distinguish these responses from genuine persistent disease. Second malignancies were recorded separately and confirmed locally with standard recommended methods.

### Statistical analysis

Recurrences are expected to be seen around 3–8 years after treatment, with the majority expected to occur before 5 years.^12^, ^13^, ^14^ Since patients were recruited to HiLo between 2007 and 2010, we decided to analyse recurrence using follow-up data up to Dec 31, 2017, to ensure that our median follow-up would be at least 5 years. Kaplan-Meier curves were obtained for time to recurrence or death, with hazard ratios (HRs) from Cox proportional hazards regressions (the assumption of proportional hazards was met). For patients with multiple recurrences, the time to first recurrence was used. Patients without recurrence were censored at their last follow-up visit. In addition to the standard statistical test for HRs, we did two non-inferiority tests. One test compared the proportion of patients with a recurrence between the radioactive iodine doses, with a margin of 5 percentage points for the difference in proportion. The other test was based on excluding an HR of 2·05 corresponding to an expected recurrence-free rate of 95% for 3·7 GBq and 90% for 1·1 GBq. These non-inferiority margins were defined post hoc (because recurrence was a secondary endpoint) but were chosen to be consistent with other similar curative cancer trials in the past, and our non-inferiority margin is in fact more stringent than that of recent non-inferiority trials that used margins of 7 percentage points.^21^, ^22^ We also did an analysis of recurrence stratified by risk of recurrence and histology at baseline. A sensitivity analysis was done by excluding patients who had a further dose of radioactive iodine. All analyses were done with Stata, version 15.1. The statistical analyses for recurrence were not prespecified in the original protocol.

This trial is registered with ClinicalTrials.gov, number NCT00415233.

### Role of the funding source

The funder of the study had no role in study design, data collection, data analysis, data interpretation, or writing of the report. The corresponding author had full access to all the data in the study and had final responsibility for the decision to submit for publication.

## Results

**Figure 1:**
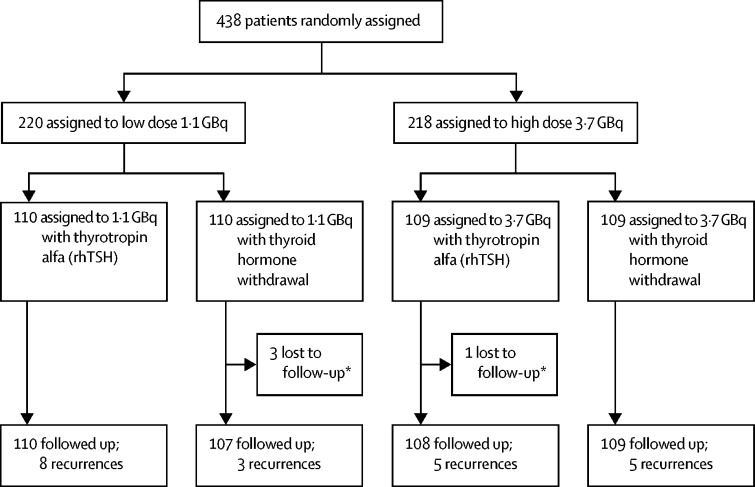
Trial profile rhTSH=recombinant human thyroid-stimulating hormone. *No information available after randomisation.

Between Jan 16, 2007, and July 1, 2010, 438 patients were randomly assigned. By Dec 31, 2017, follow-up data were available for 434 patients (four patients had no follow-up data post-ablation; figure 1). Baseline characteristics are reported in table 1. Median follow-up time was 78·4 months (range 0·3–127·3; IQR 54·4–91·3) or 6·5 (IQR 4·5–7·6) years.

**Table 1:** baseline characteristics of patients

		**Recombinant human thyroid-stimulating hormone group**		**Thyroid hormone withdrawal group**	
		1·1 GBq (n=110)	3·7 GBq (n=108)	1·1 GBq (n=107)	3·7 GBq (n=109)
Age, years		44 (34–55; 20–82)	44 (36–53; 21–76)	45 (38–53; 17–73)	43 (35–53; 18–77)
Sex					
	Women	77 (70%)	92 (85%)	79 (74%)	78 (72%)
	Men	33 (30%)	16 (15%)	28 (26%)	31 (28%)
T stage					
	T1	32 (29%)	31 (29%)	33 (31%)	32 (29%)
	T2	52 (47%)	52 (48%)	51 (48%)	50 (46%)
	T3	25 (23%)	25 (23%)	23 (22%)	27 (25%)
	Unreported	1 (1%)	0	0	0
N stage					
	N0	66 (60%)	62 (57%)	65 (61%)	63 (58%)
	N1	17 (16%)	18 (17%)	15 (14%)	17 (16%)
	Unknown	27 (25%)	28 (26%)	27 (25%)	29 (27%)
Surgery type					
	Near total	2 (2%)	0	0	1 (1%)
	Completion	62 (56%)	71 (66%)	74 (69%)	58 (53%)
	Total	46 (42%)	34 (32%)	31 (29%)	49 (45%)
	Unknown	0	3 (3%)	2 (2%)	1 (1%)

Data are n (%) or median (IQR; range).

There were 21 reported recurrences as of Dec 31, 2017 (table 2, appendix p 9). Following detailed review of reported cases, four patients (two in each group) were found to have persistent disease (all detected within 1·3 years after ablation). These four patients were included as recurrences in the analyses. Also, there was one case initially reported by the hospital as a recurrence but later thought to be a possible secondary malignancy only in the low-dose group (results below are shown with and without this case).

**Table 2:** Methods used to diagnose recurrence

	Low-dose radioactive iodine group (1·1 GBq), n=11*	High-dose radioactive iodine group (3·7 GBq), n=10*
Fine needle aspiration or biopsy	6	7
Serum thyroglobulin	7	6
Ultrasound	5	6
Diagnostic radioactive iodine scan	3	3
PET-CT scan	6	4
MRI scan	2	1

Data are n.

* Number of patients with recurrence. Each recurrence could be diagnosed with more than one method.

11 recurrences were reported in the low-dose group and ten in the high-dose group. Sites of recurrence in low-dose patients were the thyroid bed (n=6), cervical lymph nodes (n=4), para-oesophageal lymph nodes (n=1), lung metastases (n=3), soft tissue metastases (n=1), and bone metastases (n=1); and those in high-dose patients were the thyroid bed (n=6), cervical lymph nodes (n=3), and lung metastases (n=1; appendix). One patient who had 1·1 GBq (T3 stage/N0 at baseline) died from thyroid cancer, after recurrence in the thyroid bed with lung and bone metastases.

Two recurrences in the low-dose group and five in the high-dose group were seen in patients who did not have an ablation success 6–9 months after radioactive iodine ablation (when success was defined as serum thyroglobulin <2 ng/mL and scan uptake <0·1%). There were three recurrences in the low-dose group and four in the high-dose group among patients without ablation success defined using serum thyroglobulin only (ie, serum thyroglobulin ≥2 ng/mL).

The overall recurrence rate was less than 9% by 8 years, which we consider to be largely due to specialist surgeons who performed the total thyroidectomies, and careful selection of patients for the trial by the local multidisciplinary teams. However, recurrences were still being seen after 5 years of follow-up.

**Figure 2:**
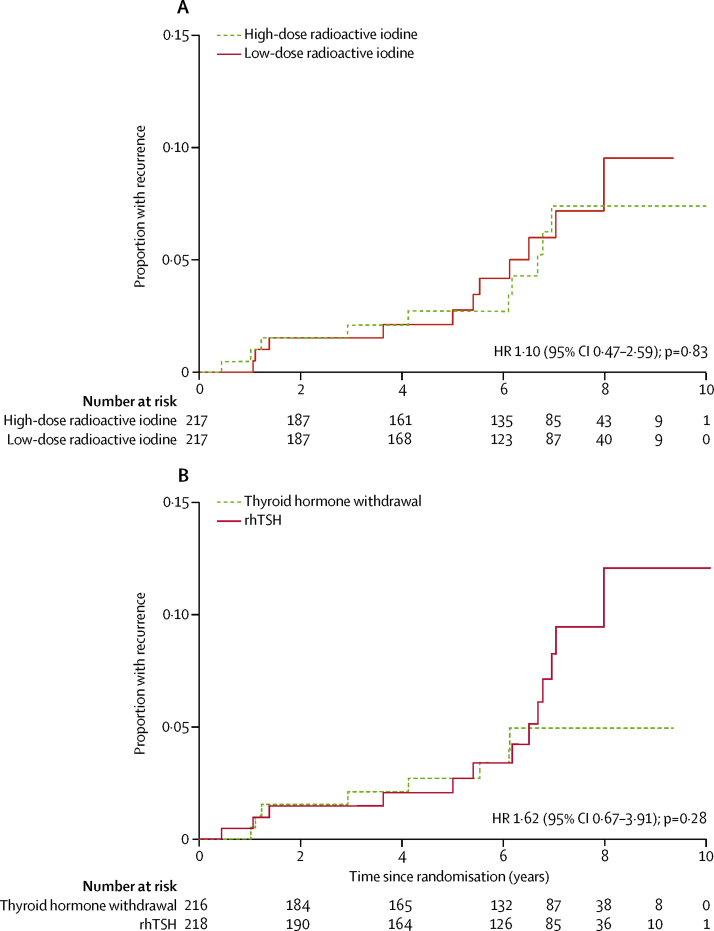
Kaplan-Meier curves showing risk of recurrence during follow-up (A) Kaplan-Meier curve of risk of recurrence according to radioactive iodine dose (3·7 GBq or 1·1 GBq). (B) Kaplan-Meier curve of risk of recurrence according to preparation for ablation with recombinant human thyroid-stimulating hormone (rhTSH) or thyroid hormone withdrawal. Kaplan-Meier curves appear to separate after about 7 years but there are only two events in total from this point. At 7 years, recurrence rates were 5·0% (95% CI 2·5–10·0) with thyroid hormone withdrawal and 8·3% (95% CI 4·5–15·3) with rhTSH, with overlapping confidence intervals.

Despite inclusion of intermediate-risk patients, recurrence rates were not materially higher in the low-dose radioactive iodine group than in the high-dose group. Kaplan-Meier cumulative recurrence rates were 1·5% with 1·1 GBq versus 2·1% with 3·7 GBq at 3 years, 2·1% versus 2·7% at 5 years, and 5·9% versus 7·3% at 7 years (HR 1·10 [95% CI 0·47–2·59]; p=0·83; figure 2). Statistical tests for non-inferiority to exclude a 5 percentage-point difference were significant for comparing two recurrence proportions (p_non-inferiority_=0·01), but not when based on the HR (p_non-inferiority_=0·08; figure 2). The Kaplan-Meier curves largely overlapped (figure 2).

After excluding the potential secondary malignancy in the low-dose group, recurrences rates were 1·5% at 3 years, 2·1% at 5 years, and 5·0% at 7 years (HR 0·99 [95% CI 0·41–2·39]; p=0·99, p_non-inferiority_=0·053 for the HR).

In a sensitivity analysis, we censored the four patients with persistent disease at the date of detection, instead of counting them as true recurrences. This analysis resulted in recurrence rates of 0·5% for 1·1 GBq versus 1·1% for 3·7 GBq at 3 years, 1·1% versus 1·7% at 5 years, and 4·9% versus 6·3% at 7 years (HR 1·13 [95% CI 0·43–2·92]; p=0·81).

Examination of the proportions of recurrences with respect to T stage and N stage provided additional evidence that 1·1 GBq was not associated with more recurrences than 3·7 GBq, even among patients with T3 tumours (table 3).

**Table 3:** Recurrence by T stage and N stage at baseline (with AJCC/UICC TNM 6th edition)

	**Low-dose radioactive iodine group**	**High-dose radioactive iodine group**
T1	3/65 (5%)	2/63 (3%)
T2	5/103 (5%)	5/102 (5%)
T3	3/48 (6%)	3/52 (6%)
N0	8/131 (6%)	4/125 (3%)
N1	2/32 (6%)	6/35 (17%)
Nx	1/54 (2%)	0/57 (0%)

AJCC/UICC=American Joint Committee on Cancer/Union for International Cancer Control. p=0·98 for the association between radioactive iodine dose and recurrence rate, stratified by either T or N stage. T stage unreported in one patient.

Results excluding the potential secondary malignancy are presented in the appendix (pp 4–6).

Among the 147 intermediate-risk patients (defined as either T3 or N1 according to the ATA 2015 risk classification system^8^) there were 12 recurrences (five [7·1%] of 70 in the low-dose group and seven [9·1%] of 77 in the high-dose group; p=0·67). In the remaining 287 low-risk patients there were nine recurrences (six [4·1%] of 147 in the low-dose group and three [2·1%] of 140 in the high-dose group; p=0·35).

A further dose of radioactive iodine (usually >3 GBq, mean dose approximately 5 GBq) was administered to 22 (10%) of 217 patients in the low-dose (1·1 GBq) group and nine (4%) of 217 in the high-dose (3·7 GBq) group (p=0·02), because of thyroid remnant in 16 patients in the low-dose group and five in the high-dose group (no re-ablations were done for recurrence according to information provided by the centres). These data were nearly the same as those in the original report (21 patients in the 1·1 GBq group *vs* nine in the 3·7 GBq group); no other additional doses were reported by the centres. Some of these further doses were due to overtreatment by some clinicians for faint temporary neck uptake following low-dose radioactive iodine treatment, which is of no significance and is well known within routine practice to disappear with time. In a sensitivity analysis excluding the 31 patients with further ablations, six recurrences were reported in the low-dose group and seven in the high-dose group, with recurrence rates of 1·1% for 1·1 GBq versus 1·1% for 3·7 GBq at 3 years, 1·1% versus 1·7% at 5 years, and 3·7% versus 5·5% at 7 years (HR 0·92 [95% CI 0·31–2·73]; p=0·87).

A confirmed second malignancy occurred in seven patients, 6 months to 7 years after ablation: in three patients in the low-dose group (plus the additional case mentioned previously, which was thought to be leiomyosarcoma or renal cancer) and in four in the high-dose group. Two left-sided breast cancers and one squamous cell carcinoma of the floor of the mouth were reported in the low-dose group. In the high-dose group, there was one squamous cell carcinoma (unknown location), one bladder cancer, one mucosa-associated lymphoid tissue lymphoma, and one myelodysplastic syndrome. The second malignancy for one patient occurred after their thyroid cancer recurrence.

When comparing patients randomly assigned to be prepared for ablation with either rhTSH or thyroid hormone withdrawal, cumulative recurrence rates were 1·5% for rhTSH versus 2·1% for thyroid hormone withdrawal at 3 years, 2·1% versus 2·7% at 5 years, and 8·3% versus 5·0% at 7 years (HR 1·62 [95% CI 0·67–3·91], p=0·28; figure 2). The Kaplan-Meier curves overlap for most of the follow-up. Tests for non-inferiority were not significant for comparisons of two proportions (p=0·09) or for excluding an HR of 2·05 (p=0·30). Furthermore, the 5-year recurrence rate was 3·2% with 1·1 GBq and rhTSH versus 4·5% with 3·7 GBq and thyroid hormone withdrawal (HR 1·56 [95% CI 0·51–4·76]; p=0·44), although there were only 13 events among 219 patients for this comparison. The appendix (p 7) shows Kaplan-Meier curves specifically for the comparison of 1·1 GBq with rhTSH versus 3·7 GBq with thyroid hormone withdrawal, as well as the comparison of the four modalities of ablation (1·1 GBq with rhTSH, 1·1 GBq with thyroid hormone withdrawal, 3·1 GBq with rhTSH, and 3·7 GBq with thyroid hormone withdrawal). No clear differences were seen between these modes of ablation.

We also found no material difference in recurrence rates between patients with different histologies at diagnosis (appendix p 8). In 229 patients with papillary thyroid cancer there were ten recurrences: four (3·4%) of 116 were given the low dose and six (5·3%) of 113 given the high dose. Among 171 patients with follicular and Hürthle cell cancers there were seven recurrences: in five (6·0%) of 83 patients given the low dose and two (2·3%) of 88 given the high dose. The HR for comparison of these two histological groups was 1·01 (95% CI 0·38–2·66; p=0·98). Histology was not reported for 34 patients, in whom there were four recurrences.

## Discussion

Long-term follow-up data from the HiLo randomised trial show that the low administered radioactive iodine dose is not associated with a higher risk of recurrence than the high dose, and nor is the risk of recurrence higher with rhTSH than with thyroid hormone withdrawal. The proportion of patients with recurrence in this study (21 [5%] of 434) is in line with the recurrence rate expected for low-risk and intermediate-risk patients with differentiated thyroid cancer.^8^ These results extend our earlier conclusions that low-dose radioactive iodine is non-inferior to high-dose radioactive iodine in terms of ablation success rate in selected patients with low-risk and intermediate-risk differentiated thyroid cancer.^5^, ^6^ Indeed, ablation success is sometimes considered a marker of recurrence or disease-related mortality.^23^ Recurrence has several consequences for patients, and studies suggest that even though patients with differentiated thyroid cancer have an excellent prognosis, several feel fear or anxiety over the potential for recurrence.^24^, ^25^

Recurrences were still seen in HiLo beyond 5 years (ie, the Kaplan-Meier curves in figure 2 were not flattening out). In some centres in routine practice, patients are discharged from the clinic at around 5 years, under the assumption that recurrences will be rare after this time. Indeed, in HiLo there were 134 patients (72 in the low-dose group and 62 in the high-dose group) without recurrence who were expected to have had a last clinic date more than 5 years after their 6–9-month ablation assessment scan, but did not; many of these patients might have been discharged back to primary care. Our findings suggest that slightly longer follow-up might be of value in some cases, which has practical significance for clinicians treating patients for thyroid cancer. A simplified surveillance protocol with the use of serum thyroglobulin alone, which is easy and inexpensive, might be considered after 5 years of follow-up for some or even all patients. Variations in national follow-up guidelines and in their implementation by individual clinicians, inclusion of intermediate-risk patients in HiLo, and variation in serum thyroglobulin assay sensitivity in different centres might explain the difference in longer-term recurrences seen in HiLo and by others. Nevertheless, our findings are consistent with those of another multicentre study in Italy in which three (23%) of 13 recurrences were observed at 5–8 years of follow-up.^26^

ESTIMABL1, the other randomised trial published alongside HiLo, reported no noticeable difference in outcomes in low-risk patients with differentiated thyroid cancer.^11^ After a median follow-up of 5·4 years, 11 of 726 patients had either persistent structural disease, serum thyroglobulin greater than 1 ng/mL, or indeterminate findings on neck ultrasonography without biopsy; of these, six had received 1·1 GBq and five had 3·7 GBq, including two cases of lymph-node recurrence.

Key strengths of the HiLo trial were the longer follow-up (6·5 years) and hence more recurrences (n=21), which were clinically confirmed with UK guidelines. A major design difference between ESTIMABL1 and HiLo was that ESTIMABL1 only enrolled low-risk patients (only two of the 752 patients had T3 disease), whereas HiLo enrolled intermediate-risk patients (100 of 438 had T3 disease). Therefore, only HiLo showed recurrence data specifically for T3 patients and showed that there was no material difference between low-dose and high-dose radioactive iodine. Inclusion of these intermediate-risk patients in HiLo is therefore of particular interest to the thyroid cancer community. We also provide Kaplan-Meier curves for recurrence, allowing clinicians and guideline authors to see when recurrences occurred and that the curves overlap. Finally, the multicentre design of HiLo increases the generalisability of the findings.

The main limitation of our study was that it was not powered to assess recurrence (a secondary outcome), which would have required a much larger trial. Therefore, although the test for non-inferiority based on comparing proportions was significant, the test based on the HR was not (p=0·08). However, the Kaplan-Meier curves overlapped throughout, strengthening our conclusions. Another limitation is that there was no standard follow-up schedule specified in the trial protocol because this would have required additional financial resources for hospitals to adhere to. UK centres follow standard guidelines from NICE and the BTA, but these guidelines are based on low-quality evidence about long-term follow-up, so there was some expected variability in the use of diagnostic tests across the centres when identifying recurrences in HiLo.

Before ESTIMABL1 and HiLo, the evidence for recurrence rates after radioactive iodine came only from observational studies. Although some individual studies reported higher recurrence rates with the low dose, two systematic reviews conducted up to 2008^12^, ^13^ concluded that there was little or no difference in either thyroid cancer recurrence or mortality in low-risk patients, among those with or without radioactive iodine ablation. This finding was confirmed by the systematic review of Lamartina and colleagues,^14^ which updated previous reviews by including studies published in 2008–14. Later studies are likely to be more reliable because of the increased sensitivity of modern tests and diagnostic methods for finding and confirming thyroid cancer recurrence. Five recent observational studies have compared low-dose and high-dose radioactive iodine (appendix p 11).^15^, ^16^, ^17^, ^18^, ^19^ In the largest of these (970 patients and 65 recurrences), the crude proportion of patients with a recurrence was lower in those who had 3·0 GBq or lower (four [2·6%] of 153) than in those who had greater than 3·0G Bq (61 [7·5%] of 817), even though the high-dose group had more patients with positive nodes (p=0·34 from the multivariable analysis). In two studies (with 225 and 204 patients), no differences were shown (two [2·4%] of 85 recurrences at the low dose versus three [2·1%] of 140 at the high dose, p=0·87; and seven [8·7%] of 80 at the low dose versus seven [5·6%] of 124 at the high dose, p=0·57). The fourth study reported no recurrences by 7 years in 176 patients. The fifth study (comprising 698 low-risk patients) also found no difference in recurrence rates overall, but hinted at a higher thyroid cancer mortality in the subgroup of patients aged 45 years or older who had 0·6–2·0 GBq (compared with those who had >2·0 GBq to 7·8 GBq); however, this analysis was based on seven events.^19^ An important limitation of these observational studies is that they were single-centre studies (except for one that comprised two hospitals^18^), in contrast to our national randomised multicentre trial (29 centres). Large, recent observational studies of low-risk and intermediate-risk patients with differentiated thyroid cancer have compared recurrence between patients who did or did not have radioactive iodine ablation.^27^, ^28^, ^29^, ^30^, ^31^, ^32^, ^33^, ^34^ Although these studies have variable follow-up times, they too have low rates of recurrence (<10% over 5–10 years; appendix p 12). None of these published studies shows a clear detriment to patients if they did not have ablation, providing further support for the findings from HiLo, because these studies are expected to show larger differences in recurrence rates, if they exist, than those comparing high and low doses of radioactive iodine.

The combination of recent large observational studies and long-term follow-up of two randomised trials (HiLo and ESTIMABL1) represents comprehensive and definitive evidence that low-dose radioactive iodine is an acceptable therapy for patients with differentiated thyroid cancer. The next step is to show whether radioactive iodine ablation can be avoided completely in patients at very low risk. Two large prospective trials are ongoing, the IoN trial (NCT01398085) and ESTIMABL2 (NCT01837745), both of which involve randomly assigning selected low-risk patients to have either 1·1 GBq radioactive iodine ablation or no radioactive iodine ablation, with disease-free survival as a major endpoint.

In 2012, the European Medicines Agency extended the licence indication for rhTSH, on the basis of the results for ablation success, allowing it to be used with 1·1 GBq. But the agency requested long-term data about recurrences be made available at a later date. Data from the HiLo trial (showing Kaplan-Meier curves for recurrence) provide this evidence to further support the use of rhTSH with low-dose radioactive iodine.

In conclusion, after a median follow-up of more than 6 years in HiLo, the recurrence rate was similar between patients who had low-dose radioactive iodine ablation and those who had the high dose (and between those prepared for ablation with rhTSH and those who underwent hormone withdrawal). Together with the results of the ESTIMABL1 trial and contemporary observational studies, reliable evidence is now available to strengthen guidelines and further assure patients and clinicians of the benefits of using the low administered dose.
