# Supplementary material for: Recurrence after low-dose radioiodine ablation and recombinant human thyroid-stimulating hormone for differentiated thyroid cancer (HiLo): long-term results of an open-label, non-inferiority randomised controlled trial
Source: Lancet Diabetes Endocrinol. 2019 Jan;7(1):44–51. doi: 10.1016/S2213-8587(18)30306-1 (PMC6299255; doi:10.1016/S2213-8587(18)30306-1)
Supplement: Supplementary appendix [file mmc1.pdf]

THE LANCET
Diabetes & Endocrinology

Supplementary appendix

This appendix formed part of the original submission and has been peer reviewed. We post it as supplied by the authors.

Supplement to: Dehbi H-M, Mallick U, Wadsley J, Newbold K, Harmer C, Hackshaw A . Recurrence after low-dose radioiodine ablation and recombinant human thyroid-stimulating hormone for differentiated thyroid cancer (HiLo): long-term results of an open-label, non-inferiority randomised controlled trial. *Lancet Diabetes Endocrinol* 2018; published online Nov 27. http://dx.doi.org/10.1016/S2213-8587(18)30306-1.

Appendix

Content **Page**

Clinicians and recruiting centres 2

Inclusion/exclusion criteria 3

Appendix Figure 1 4

Appendix Table 1 5

Appendix Table 2 6

Appendix Figure 2 7

Appendix Figure 3 8

Appendix Table 3 9

Appendix Table 4 11

Appendix Table 5 12

References 15

Clinicians and recruiting centres, by alphabetical order of hospital name

Sarah Jeffries (Addenbrooke's Hospital); Matthew Beasley (Bristol Haematology and Oncology Centre); Abdel Abdel-Hamid (Castle Hill Hospital);Roger Owen (Cheltenham General Hospital); Beng Yap (Christie Hospital NHS Trust); Michael Williams (Cumberland Infirmary); Elena Macias (East Kent Hospitals University NHS Foundation Trust); Ujjal Mallick (Freeman Hospital); Simon Gollins (Glan Clwyd Hospital); Mohan Hosahalli (Guys Hospital); Christopher Scrase (Ipswich Hospital NHS Trust); John Hardman (James Cook University Hospital); Irene Peat (Leicester Royal Infirmary); Nick Rowell (Maidstone & Tunbridge Wells); Catherine Lemon (Mount Vernon Hospital); Caroline Brammer (New Cross Hospital, Wolverhampton); Tom Roques (Norfolk & Norwich University Hospital); Roy Matthews (Northampton General Hospital); Perric Crellin (Poole Hospital NHS Foundation Trust); Rengarajan Vijayan (Royal Derby Hospitals); Andrew Goodman (Royal Devon & Exeter Hospital); Christopher Nutting (Royal Marsden Hospital); Stephen Whitaker (Royal Surrey County Hospital); Joanna Simpson (Royal Sussex County Hospital); Georgina Gerrard (St James's University Hospital); Anna Cassoni (University College London Hospital Trust); Arshad Jamil (University Hospital North Staffordshire); Laura Moss (Velindre Cancer Centre); Jonathan Wadsley (Weston Park Hospital).

Inclusion / exclusion criteria

Inclusion:

- Histological confirmation of differentiated thyroid carcinoma
- Patients with tumour stage pT1-T3; NX, N0 or N1, M0 (TNM 6th edition 2002)
- Patients who have undergone total thyroidectomy with or without lymph node dissection
- Patients who require radioiodine ablation
- Age 16-80 years
- WHO performance status 0 - 2 (self caring)
- All known tumour resected (R0)

Exclusion:

- Aggressive variants including tall cell, insular, poorly differentiated and diffuse sclerosing; anaplastic and medullary carcinoma
- Patients who have a contrast CT scan up to 3 months before ablation
- Patients for whom recombinant human TSH requirement is mandatory
- Patients who have severe co-morbid conditions (e.g. unstable angina, recent heart attack or stroke, severe labile hypertension, dementia, on dialysis, with tracheostomy needing care, learning difficulties and anybody who may not be able to comply with radiation protection issues or need frequent nursing/medical supervision which puts staff at risk of unacceptable radiation exposure)
- Previous malignancies with limited life expectancy likely to interfere with the patient's ability to be able to comply with treatment and/or follow up
- Pregnant women or women who are breastfeeding. A pregnancy test should be offered if necessary
- Patients with stage pT4 or M1 (if detected clinically or by other investigations)
- Previous 131I or 123I pre-ablation scan
- Previous treatment for thyroid cancer (except surgery)

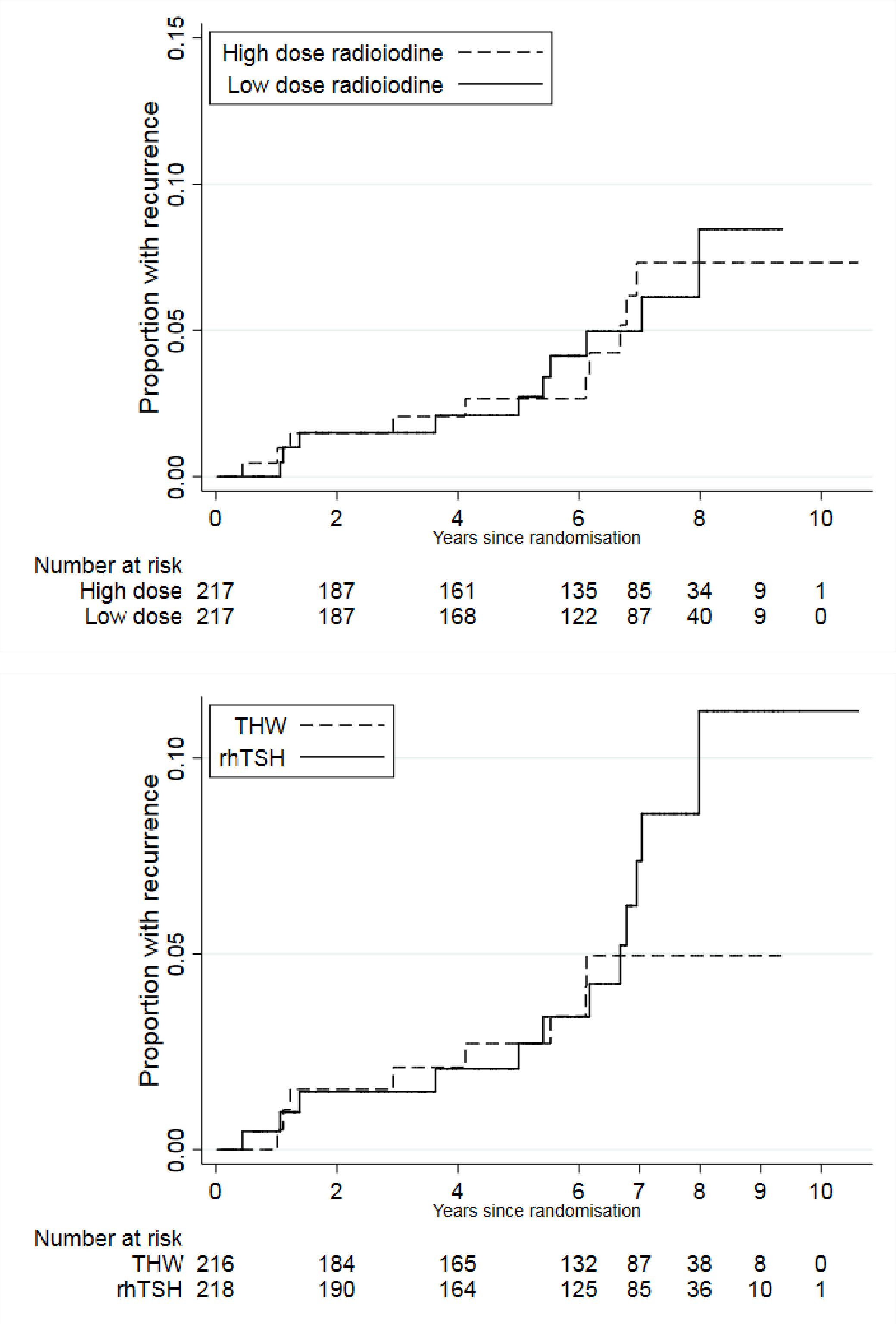

Appendix Figure 1: Excluding the potential secondary malignancy, risk of recurrence according to RAI dose (3.7 or 1.1 GBq) upper panel; or preparation for ablation using recombinant human thyroid-stimulating hormone (rhTSH) or thyroid hormone withdrawal (TWH) lower panel. In the lower panel, the Kaplan-Meier curves appear to separate after about 7 years but there are only 2 events in total from then. At 7 years, recurrence rates were 5.0% (95% CI 2.5-10.0) with THW and 7.4% (95% CI 3.9-14.1) with rhTSH, with greatly overlapping confidence intervals.

Appendix table 1: Excluding the potential secondary malignancy, methods used to diagnose recurrence. Each recurrence may be diagnosed with more than one method.

		Methods of diagnoses of recurrence					
Arm	Number of patients with recurrence	Fine needle aspiration / biopsy	Serum thyroglobulin	Ultrasound	Diagnostic RAI scan	CT/PET scan	MRI scan
Low dose RAI (1.1 GBq)	10	6	7	5	3	5	2
High dose RAI (3.7 GBq)	10	7	6	6	3	4	1

Appendix Table 2: Excluding the potential secondary malignancy, recurrence by T- and N- stage at baseline (using AJCC/UICC* TNM 6th edition)

	T1	T2	T3	p-value**
Low dose RAI	3.1% (2/65)	4.9% (5/103)	6.2% (3/48)	0.84
High dose RAI	3.2% (2/63)	4.9% (5/102)	5.8% (3/52)	
	N0	N1	Nx	p-value**
Low dose RAI	6.1% (8/131)	6.2% (2/32)	0.0% (0/54)	0.83
High dose RAI	3.2% (4/125)	17.1% (6/35)	0.0% (0/57)	

* American Joint Committee on Cancer / Union for International Cancer Control

** for the association between RAI dose and recurrence rate, stratified by either T or N stage

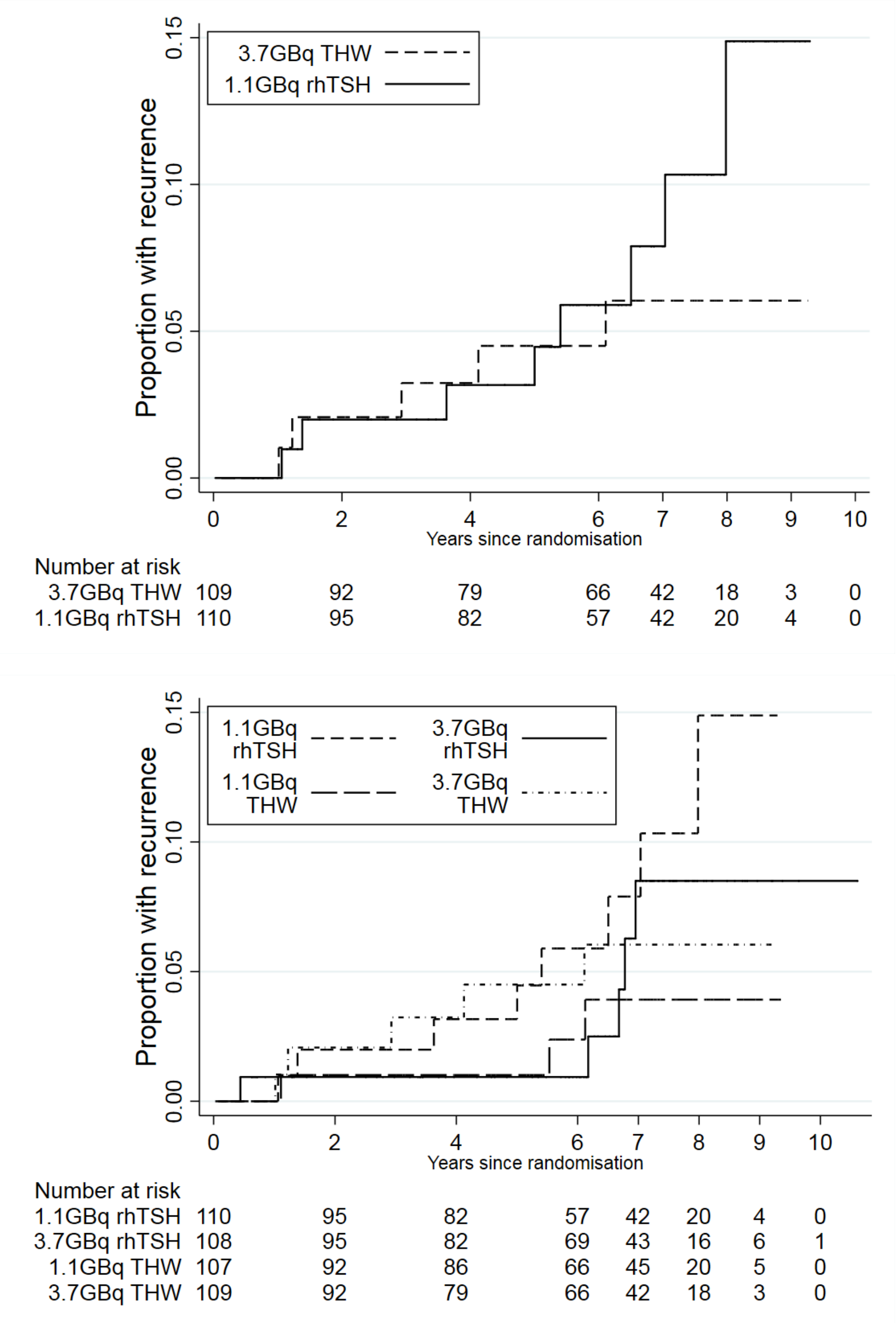

Appendix Figure 2: Risk of recurrence according to recombinant human thyroid-stimulating hormone (rhTSH) with low dose RAI or thyroid hormone withdrawal (THW) with high dose RAI (upper panel). This analysis is based on 219 patients with 13 events in total. The curves only separate after 6 years but there are 4 events only from here in total. Risk of recurrence according to the four modalities of ablation (1.1 GBq with rhTSH, 1.1 GBq with THW, 3.1 GBq with rhTSH and 3.7 GBq with THW) (lower panel).

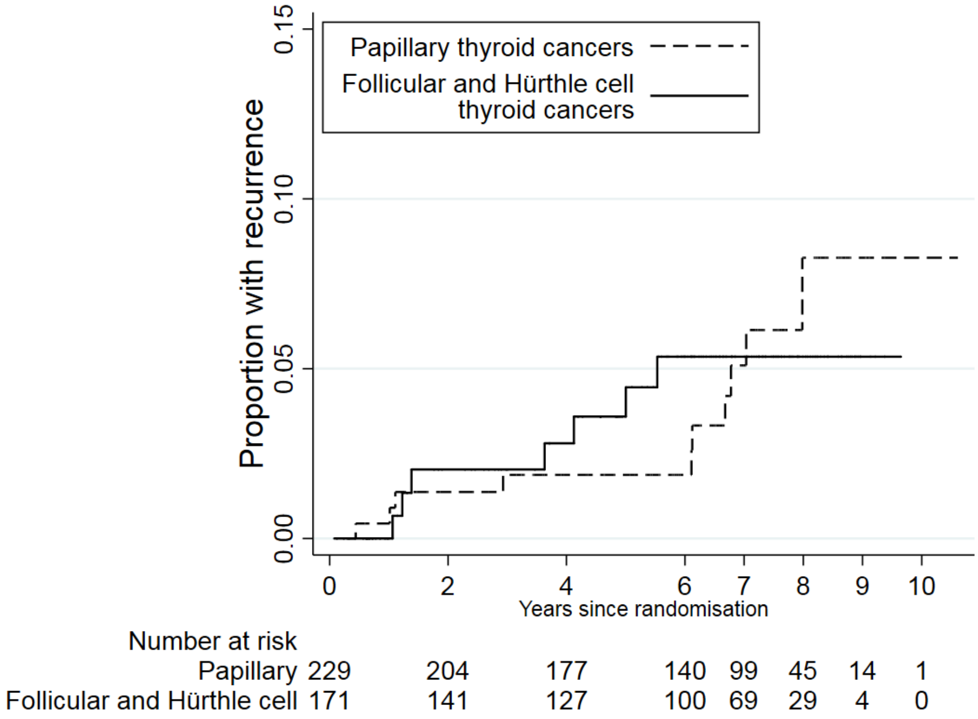

Appendix Figure 3: Risk of recurrence according to cancer histology at diagnosis. The hazard ratio is 1.01 [95% CI 0.38-2.66, p-value=0.98].

Appendix Table 3: Methods used to diagnose each reported recurrence and patient baseline characteristics (age, sex and T and N stage).

								Site of recurrence					Methods of diagnosis of recurrence					
Age	Sex	T	N	Histology	Tg at 6-9 months	Ablation success: ultrasound/Tg*	Time to recurrence [years]	Thyroid bed	Lymph nodes	Lung	Soft tissue	Bone	RAI	Tg	Ultra sound	CT/ PET	MRI	FNA
**High dose (3.7 GBq)**																		
36	F	3	1	papillary	7.9	failure	1.0	Y							Y			Y
34	F	2	1	papillary	0.5	success	6.6	Y					Y	Y				
37	F	1	1	papillary	1.4	success	6.7	Y					Y	Y				
39	F	2	0	follicular	53.9	failure	1.1[##]	Y					Y	Y	Y			Y
40	F	1	0	papillary	0.5	success	6.0	Y						Y***		Y		Y
40	M	2	0	unknown	2.8	failure	6.9		Y						Y	Y		Y
37	M	2	1	papillary	n.a.	not assessable[#]	0.4[##]		Y					Y	Y			Y
21	F	3	0	unknown	0.7	success	6.1	Y							Y			Y
71	F	2	1	papillary	n.a	failure	2.9			Y				Y		Y		
59	M	3	1	follicular	123.4	failure	4.1		Y						Y	Y	Y	Y
**Low dose (1.1 GBq)**																		
32	M	1	1	papillary	0.5	success	7.0	Y	Y				Y	Y				
65	M	2	0	papillary	0.5	success	7.9		Y**					Y		Y		Y****
52	F	1	X	unknown	0.5	success	6.4			Y	Y					Y~		
22	F	2	1	papillary	n.a.	not assessable[#]	6.1		Y					Y	Y		Y	Y****
42	M	3	0	Hürthle cell	279.4	failure	1.3[##]	Y						Y	Y			Y
56	F	1	0	unknown	0.8	success	5.4	Y					Y	Y				
47	M	2	0	papillary	0.5	success	1.0[##]	Y					Y					
54	F	2	0	Hürthle cell	n.a.	not assessable[#]	3.6		Y					Y	Y	Y		Y
68	M	3	0	follicular	0.7	success	5.5			Y				Y***		Y		
57	M	3	0	Hürthle cell	376.8	failure	1.0	Y	Y	Y		Y			Y	Y	Y	Y
67	F	2	0	follicular	21.1	success	4.9	Y							Y	Y		Y

n.a. not available; ***** ablation success defined as Tg<2 ng/ml <u>and</u> scan <0.1% uptake; [#] because ultrasound and Tg were not both available; [##] on review was considered to be persistent disease; ** para-oesophageal lymph nodes; all other lymph node recurrences were cervical lymph nodes; *** thyroglobulin antibody present; **** biopsy; ~ was identified as potential secondary malignancy; FNA: fine needle aspiration

Appendix Table 4: Evidence from recent observational studies (published since 2008) of the effect of low versus high RAI on recurrence in low or intermediate risk patients with well-differentiated thyroid cancer.

Reference; country	No. of patients	Patients included	RAI dose	Approximate median follow-up	Recurrence rates for low vs high dose*
Kruijff et al 2013[1]; Australia	970	Papillary with T1-T3	≤ 3 vs > 3 GBq	5 years	4/153 (2.6%) vs 61/817 (7.5%); p=0.04
Verburg et al 2014[2]; Germany	698	Papillary and follicular with T1-T2, without lymph node or distant metastases	0.6-2 GBq (low) vs 2-3 GBq (middle) vs 3-7.8 GBq (high)	Range 6-17 years	Low vs middle vs high:<br><br>Age <45 years:<br>0 vs 0.9 vs 2.3% (p=0.62)#<br><br>Age ≥ 45 years:<br>3.6 vs 5.6 vs 5.4% (p=0.92)#
Castagna et al 2013[3]; Italy	225	Intermediate risk patients**	1.1-1.8 vs ≥3.7 GBq	5.5 years	2/85 (2.4%) vs 3/140 (2.1%); p=0.87
Jeong et al 2017[4]; South Korea	204	Intermediate risk patients##	1.1 vs 3.7-5.55 GBq	10 years	7/80 (8.8%) vs 7/124 (5.6%); p=0.57
Han et al 2014[5]; South Korea	176	Tumour size ≤2cm	1.1 vs 5.5 GBq	7.2 years	No recurrences in either group

* P-value calculated using exact test of proportions with continuity correction based on observed recurrence rates

# P-value reported in the paper, based on log-rank tests

** According to ATA 2009[6]; ## According to ATA 2015[7]

Appendix Table 5: Summary of evidence from observational studies published in 2008 or after of the effect of RAI on recurrence in low or intermediate risk patients with well-differentiated thyroid cancer (studies ordered by number of patients).

Reference	Study size	Cancer	RAI treatment	Approximate median follow-up	Results for low vs high dose, or no RAI vs RAI
**Low versus high dose RAI ablation**					
Kruijff et al.[1]	970	PTC with T1-T3	≤ 3 GBq<br>vs<br>> 3 GBq	5 years	Recurrences:<br>Combined<br>4/153 (2.6%) vs 61/817 (7.5%); p=0.04<br><br>T1 tumours: 2/100 (2%) vs 14/379 (4%); p=0.54<br>T2 tumours: 0/24 (0%) vs 10/140 (7%); p=0.36<br>T3 tumours: 2/29 (7%) vs 37/298 (12%); p=0.55
Verburg et al.[2]	698	PTC and FTC with T1-T2 without lymph node or distant metastases	0.6-2 GBq (low)<br>vs<br>2-3 GBq (middle)<br>vs<br>3-7.8 GBq (high)	range: 6-17 years	Recurrences after 15 years of follow-up:<br>- Low vs middle vs high:<br>Age <45 years:<br>0 vs 0.9 vs 2.3% (p=0.62)<br>Age ≥ 45 years:<br>3.6 vs 5.6 vs 5.4% (p=0.92)<br><br>DTC-related mortality after 15 years of follow-up:<br>- Low vs middle vs high:<br>Age <45 years:<br>0 vs 1.1 vs 0% (p=0.38)<br>Age ≥ 45 years:<br>16.1 vs 0.8 vs 7.2% (p=0.004)
Castagna et al.[3]	225	IR DTC patients	1.1-1.8 GBq<br>vs<br>≥ 3.7 GBq	5.5 years	Recurrences: 2/85 (2.4%) vs 3/140 (2.1%); p=0.87
Jeong et al.[4]	204	IR DTC patients	1.1 GBq	10 years	Recurrences: 7/80 (8.8%) vs 7/124 (5.6%); p=0.57

			vs<br>3.7-5.55 GBq		
Han et al.[5]	176	Tumour size ≤2cm	1.1 GBq<br>vs<br>5.5 GBq	7.2 years	No recurrences in either group
**No RAI versus RAI ablation**					
Kwon et al.[8]	1932	PMC	3-5.5 GBq	8.3 years	HR (multivariate) for recurrence (RAI vs no RAI):<br>2.02 (95% CI: 0.65 - 6.25); p=0.22
Buffet et al.[9]	1669	PMC, incl. pT1a, pT3 and Nx, N1	3.7 GBq	4.7 years	10-year recurrence rate: 10% vs 6%; p=0.96<br><br>In patients with pT3, RAI associated with recurrence: HR 0.97 (95% CI 0.95-0.99); p<0.002
Schvartz et al.[10]	1298	LR PTC or FTC, and pT1 and pT2 without Nx	3.7 Gbq	10.3 years	10-year OS: 95.8% vs 94.6% - HR 0.69 (95% CI 0.37-1.29); p=0.24<br><br>10-year DFS: 93.1% vs 88.7% - HR 0.73 (95% CI 0.43-1.25); p=0.26
Hay et al.[11]	900	LR PMC	Range of doses	17.2 years (mean)	512 node-negative patients: 0.6% recurrence vs. 0%; p=0.79<br><br>253 node-positive patients: no impact of RAI on recurrence at local (p=0.81) and distant sites (p=0.68)
Kim et al.[12]	704	LR and IR PMC, incl. N1a and N1b	Range of doses	5.3 years	Recurrences: 0/126 (0%) vs 6/578 (1%); p=0.17
Ross et al.[13]	611	PMC, incl. Nx and N1	Range of doses	2.8 years (mean)	Node-positive patients: 3 recurrences (11%) vs 18 (17%); p=ns<br><br>Node-negative patients: 7 patients (2%) vs 10 (6%); p=0.05
Nixon et al.[14]	532	IR PTC > 45 years old, incl. pT1 and pT2, N0 and N1.<br>IR PTC ≤ 45 years old, incl. pT3 and pT4, N0 and N1	Range of doses	4.5 years	5-year RFS:<br>> 45 years old: 98% vs 96%; p=0.23<br>≤ 45 years old: 95% vs 88%; p=0.26
Pelttari et al.[15]	495	PTC or FTC stage 1 or 2 by TNM	1.1-4.4 GBq	11.6 years	Recurrences: 36/398 (9%) vs 8/97 (8%); p=0.80

Abbreviations:

PTC	Papillary thyroid carcinoma		RR	Relative risk
DTC	Differentiated thyroid cancer		RFS	Recurrence-free survival
ETE	Extrathyroidal extension		HR	Hazard ratio
PMC	Papillary thyroid microcarcinoma		LR	Low risk (of recurrence)
FTC	Follicular thyroid carcinoma		IR	Intermediate risk (of recurrence)

References

1. Kruijff S, Aniss AM, Chen P, Sidhu SB, Delbridge LW, Robinson B, et al. Decreasing the dose of radioiodine for remnant ablation does not increase structural recurrence rates in papillary thyroid carcinoma. Surgery. 2013;154(6):1337-44

2. Verburg FA, Mader U, Reiners C, Hanscheid H. Long-term survival in differentiated thyroid cancer is worse after low-activity initial post-surgical 131I therapy in both high- and low-risk patients. The Journal of clinical endocrinology and metabolism. 2014;99(12):4487-96

3. Castagna MG, Cevenini G, Theodoropoulou A, Maino F, Memmo S, Claudia C, et al. Post-surgical thyroid ablation with low or high radioiodine activities results in similar outcomes in intermediate risk differentiated thyroid cancer patients. European journal of endocrinology / European Federation of Endocrine Societies. 2013;169(1):23-9

4. Jeong JH, Kong EJ, Jeong SY, Lee S-W, Cho IH, Ah Chun K, et al. Clinical outcomes of low-dose and high-dose postoperative radioiodine therapy in patients with intermediate-risk differentiated thyroid cancer. Nuclear medicine communications. 2017;38(3):228-33

5. Han JM, Kim WG, Kim TY, Jeon MJ, Ryu JS, Song DE, et al. Effects of low-dose and high-dose postoperative radioiodine therapy on the clinical outcome in patients with small differentiated thyroid cancer having microscopic extrathyroidal extension. Thyroid : official journal of the American Thyroid Association. 2014;24(5):820-5

6. Cooper DS, Doherty GM, Haugen BR, Kloos RT, Lee SL, Mandel SJ, et al. Revised American Thyroid Association management guidelines for patients with thyroid nodules and differentiated thyroid cancer. Thyroid : official journal of the American Thyroid Association. 2009;19(11):1167-214

7. Haugen BR, Alexander EK, Bible KC, Doherty GM, Mandel SJ, Nikiforov YE, et al. 2015 American Thyroid Association Management Guidelines for Adult Patients with Thyroid Nodules and Differentiated Thyroid Cancer: The American Thyroid Association Guidelines Task Force on Thyroid Nodules and Differentiated Thyroid Cancer. Thyroid : official journal of the American Thyroid Association. 2016;26(1):1-133

8. Kwon H, Jeon MJ, Kim WG, Park S, Kim M, Kim TY, et al. Lack of Efficacy of Radioiodine Remnant Ablation for Papillary Thyroid Microcarcinoma: Verification Using Inverse Probability of Treatment Weighting. Annals of Surgical Oncology. 2017;24(9):2596-602

9. Buffet C, Golmard JL, Hoang C, Trésallet C, Du Pasquier Fédiaevsky L, Fierrard H, et al. Scoring system for predicting recurrences in patients with papillary thyroid microcarcinoma. European Journal of Endocrinology. 2012;167(2):267-75

10. Schvartz C, Bonnetain F, Dabakuyo S, Gauthier M, Cueff A, Fieffé S, et al. Impact on Overall Survival of Radioactive Iodine in Low-Risk Differentiated Thyroid Cancer Patients. The Journal of Clinical Endocrinology & Metabolism. 2012;97(5):1526-35

11. Hay ID, Hutchinson ME, Gonzalez-Losada T, McIver B, Reinalda ME, Grant CS, et al. Papillary thyroid microcarcinoma: A study of 900 cases observed in a 60-year period. Surgery. 2008;144(6):980-8

12. Kim HJ, Kim NK, Choi JH, Kim SW, Jin S-M, Suh S, et al. Radioactive iodine ablation does not prevent recurrences in patients with papillary thyroid microcarcinoma. Clinical endocrinology. 2013;78(4):614-20

13. Ross DS, Litofsky D, Ain KB, Bigos T, Brierley JD, Cooper DS, et al. Recurrence After Treatment of Micropapillary Thyroid Cancer. Thyroid : official journal of the American Thyroid Association. 2009;19(10):1043-8

14. Nixon IJ, Patel SG, Palmer FL, et al. Selective use of radioactive iodine in intermediate-risk papillary thyroid cancer. Archives of Otolaryngology – Head & Neck Surgery. 2012;138(12):1141-6

15. Pelttari H, Laitinen K, Schalin-Jäntti C, Välimäki MJ. Long-term outcome of 495 TNM stage I or II patients with differentiated thyroid carcinoma followed up with neck ultrasonography and thyroglobulin measurements on T4 treatment. Clinical endocrinology. 2008;69(2):323-31
